# Effect of Secondary Crosslinking Time on the Interfacial Insulation Performance of Crosslinked Polyethylene/Semiconductive Shielding Layer

**DOI:** 10.3390/polym18111277

**Published:** 2026-05-22

**Authors:** Ming Hu, Hongliang Zhang, Xufei Ge, Yan Yan, Yuanhang Yang, Xiaoyan Cao, Zerui Li, Wenbo Huo

**Affiliations:** 1 College of Mechanical and Electrical Engineering, Guangdong University of Technology, Guangzhou 510006, China; hum@ztt.cn; 2 Zhongtian Technology Submarine Cable Co., Ltd., Nantong 226000, China; 3 College of Electrical Engineering, Sichuan University, Chengdu 610065, China

**Keywords:** secondary crosslinking time, XLPE/semiconductive shielding layer interface, insulation performance, flexible joint

## Abstract

To investigate the influence of secondary crosslinking time on the interfacial insulation performance between crosslinked polyethylene (XLPE) and a semiconductive shielding layer, XLPE sheets and semiconductive EVA pellets were selected. XLPE/semiconductive shielding layer interfacial specimens with secondary crosslinking times of 10 min, 15 min, 30 min, 45 min and 60 min were prepared. Polarization and depolarization current (PDC) measurements, breakdown voltage tests, peel adhesion strength evaluation and scanning electron microscopy (SEM) observations were systematically performed. The interfacial polarization current, characteristic breakdown voltage and interfacial peel adhesion strength of the specimens were obtained and analyzed. The experimental results indicate that, with increasing secondary crosslinking time, the interfacial polarization current showed an initial decrease followed by an increase, and the characteristic breakdown voltage and the interfacial peel adhesion strength showed an initial increase followed by a decrease. Further analysis suggests that an excessively long secondary crosslinking time reduces the area of the interfacial interpenetration region between XLPE and the semiconductive shielding layer, which is the primary factor responsible for the deterioration of interfacial insulation performance. The results provide experimental evidence and theoretical support for optimizing flexible joint manufacturing processes and improving their operational reliability and service lifetime.

## 1. Introduction

With the large-scale development of offshore wind power and the accelerated construction of inter-island power grid interconnection projects, submarine cables have become the key energy transmission corridors connecting far-offshore wind farms to onshore power grids. Consequently, their voltage levels continue to increase and the scale of deployment rapidly expands [1,2,3]. Crosslinked polyethylene (XLPE) cables owing to their excellent electrical insulation performance, mechanical strength and aging resistance have become the core equipment in offshore wind power transmission systems [4,5]. However, constrained by manufacturing processes and installation conditions, the length of a single submarine cable is often insufficient for long distance deployment, making intermediate joints indispensable for multi-segment connections. In this context, submarine cable flexible joints play a critical role in ensuring the safe and reliable operation of the entire transmission line. Their performance and reliability directly determine the service life and power transmission capability of submarine cable systems [6,7].

The primary function of submarine cable flexible joints is to restore the layered structural continuity of the cable materials [8,9]. During the injection molding process of flexible joints, a secondary interface formed between adjacent material layers becomes a critical and vulnerable region that governs the overall performance of the joint [10,11]. The quality of this secondary interface is jointly determined by key processing parameters such as injection temperature, pressure, and time [12,13,14,15,16]. These parameters not only affect the interfacial bonding integrity and defect density, but also significantly influence the electrical strength, mechanical properties and long-term aging resistance of the interface, which are essential for the reliable operation of submarine cable flexible joints under complex marine environments.

In recent years, most studies have focused on the secondary interface between XLPE/XLPE with extensive investigations into surface roughness control, degassing treatment and injection molding process optimization [17]. Correspondingly, systematic studies on the electrical, physicochemical, and mechanical properties of XLPE/XLPE interfaces have been reported. For instance, Meng suggested that higher surface roughness can enhance interfacial crystallinity, while extended degassing time improves the breakdown strength of XLPE/XLPE interfaces [12,15,16]. Zhang reported that increasing vulcanization pressure effectively improves the electrical performance of XLPE/XLPE interfaces [13]. In contrast, existing studies have mostly investigated the electrical behavior in the vicinity of the XLPE/semiconductive shielding layer from the perspectives of semiconductive shielding-layer material modification, suppression of space charge injection, and degradation induced by interfacial defects, whereas studies on the secondary interface between XLPE insulation and semiconductive shielding layers remain relatively limited particularly in the context of offshore wind power systems evolving toward higher voltage levels and longer transmission distances [18,19,20]. The interfacial performance between the XLPE insulation layer and the semiconductive shielding layer is crucial for electric field homogenization and partial discharge suppression [21,22,23]. However, the effects of key injection molding parameters such as temperature, pressure, and time on the microstructure and insulation performance of the XLPE/semiconductive shielding layer interface are not yet fully understood. This knowledge gap has become one of the major bottlenecks restricting the development of high performance flexible joint technologies.

To investigate the influence of secondary crosslinking time on the interfacial characteristics of XLPE/semiconductive shielding layers, XLPE/semiconductive layer secondary interface specimens with crosslinking times of 10 min, 15 min, 30 min, 45 min and 60 min are prepared in this study. A series of characterization techniques including PDC measurements, breakdown strength tests, peel adhesion strength evaluation and scanning electron microscopy (SEM) analysis are systematically conducted. By comparatively analyzing the electrical performance, interfacial adhesion behavior and microstructural features under different secondary crosslinking times, the effects of secondary crosslinking time on interfacial insulation performance, peel adhesion strength and interfacial structure are elucidated.

## 2. Materials and Methods

### 2.1. Materials and Sample Preparation

**Figure 1:**
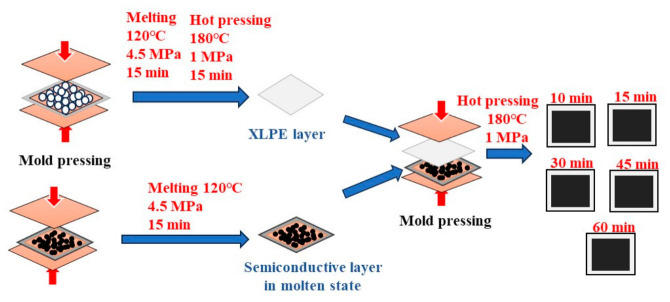
Preparation schematic diagram of XLPE/semiconductor shielding layer interface samples at different secondary crosslinking times.

XLPE/semiconductive shielding layer interface specimens with different crosslinking times were prepared in the laboratory by a flat-plate hot-pressing method, using commercial XLPE cable pellets, to simulate the manufacturing process of XLPE/semiconductive shielding layer interfaces in factory joints. A schematic illustration of the preparation procedure was shown in Figure 1. The detailed steps were described as follows.

First, a small amount of XLPE cable pellets which were composed of low-density polyethylene (LDPE, prepared by Zhongtian Submarine Cable Co., Ltd., Nantong, China) with 2.0 wt% dicumyl peroxide (DCP), and auxiliary additive (including antioxidants) was placed into a mold with dimensions of 150 mm × 150 mm × 0.2 mm. The pellets were preheated in a flat-plate vulcanizing (produced by Beijing Fuyou Co., Ltd. in Beijing, China) press at 120 °C under a pressure of 4.5 MPa for 15 min. Subsequently, the temperature was increased to 180 °C, and crosslinking was conducted at a pressure of 1 MPa for 15 min. After cooling to room temperature, a sheet (denoted as sample A) simulating the recovered insulation layer of a cable joint was obtained, with dimensions of 150 mm × 150 mm × 0.2 mm. Second, a small amount of semiconductive shielding pellets (main raw material is EVA, with a medium vinyl acetate (VA) content of 20–28% and a melt index (MI) of 2–10 g/10 min) was placed into a 150 mm × 150 mm × 0.2 mm mold and preheated at 120 °C under 4.5 MPa for 15 min using the flat plate vulcanizing press. The previously prepared XLPE insulation recovery layer sheet (sample A) was then placed on top of the preheated semiconductive material. Another mold with dimensions of 150 mm × 150 mm × 0.4 mm was placed above the assembly to maintain the thickness of the secondary crosslinked semiconductive interface at approximately 0.4 mm. Secondary crosslinking was subsequently carried out at 180 °C under a pressure of 1 MPa for 10 min, 15 min, 30 min, 45 min and 60 min, respectively. As a result, XLPE/semiconductive shielding layer interface specimens with different crosslinking times were obtained. The final specimen dimensions were 150 mm × 150 mm × 0.4 mm, and the samples were labeled as #10, #15, #30, #45 and #60 according to their secondary crosslinking times.

Finally, the prepared XLPE/semiconductive shielding layer interface specimens were subjected to a degassing treatment in a vacuum oven at 70 °C for 48 h to remove crosslinking by products as thoroughly as possible, thereby minimizing their influence on subsequent electrical and mechanical measurements.

### 2.2. Polarization and Depolarization Current (PDC) Measurement

The basic principle of the polarization and depolarization current (PDC) measurement is as follows [24,25]. A DC polarization voltage is first applied to the dielectric specimen to induce polarization and conduction processes within the material. After the voltage is removed, the decay behavior of the depolarization current is recorded. During these two stages, dipoles inside the dielectric undergo orientation and relaxation in response to the external electric field. The insulation condition of the dielectric can therefore be evaluated through the characteristics of the polarization and depolarization current responses.

**Figure 2:**
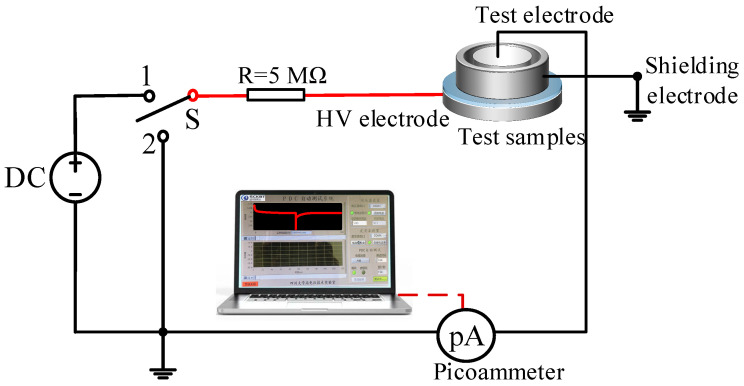
Schematic diagram of polarization-depolarization measuring.

For the flat insulating specimens used in this study, a three-electrode measurement system with a guard ring electrode configuration was employed. The detailed electrical connections, including the grounded electrode, high voltage electrode and guard ring shielding electrode are illustrated in Figure 2. This electrode configuration effectively suppresses surface leakage currents, thereby improving the accuracy and reliability of the PDC measurements.

Considering the relatively small thickness of the specimens, a polarization voltage of 1 kV was applied and the polarization duration was set to 120 s. This ensured sufficient establishment of the polarization process while avoiding dielectric breakdown caused by excessive electric stress, enabling the acquisition of reliable dielectric response data within a safe operating range. The measurement was repeated at least five times to minimize random interference during the experiment. The polarization and depolarization currents are expressed by Formulas (1) and (2), respectively:

(1) $$i_{pol}\left(t\right)=C_{0}U_{0}\left[\frac{\sigma }{\varepsilon_{0}}+f\left(t\right)\right]$$

(2) $$i_{depol}\left(t\right)=C_{0}U_{0}\left[f\left(t\right)-f\left(t+t_{c}\right)\right]$$

where *U*_0_ is the constant polarization voltage. *C*_0_ is the geometric capacitance of the dielectric. σ is the DC conductivity. *ε*_0_ is the vacuum permittivity. *t* is the measurement time, *t*_c_ is the duration of the applied polarization voltage and *f*(*t*) is the dielectric response function.

The DC conductivity, which directly reflects the insulation condition of the cable material [26,27], can be calculated using Formula (3):

(3) $$\sigma =\frac{\varepsilon_{0}\left(ave(i_{pol-end})-ave(i_{depol-end})\right)}{C_{0}U_{0}}$$

where ave(*i_pol-end_*) and ave(*i_depol-end_*) represent the steady state average values of the polarization current and depolarization current at the end of the measurement, respectively.

Interfacial polarization arises from differences in electrical conductivity and relative permittivity between the two materials forming the interface. The interfacial polarization current can therefore serve as an effective indicator of interfacial insulation performance. The interfacial polarization current is calculated according to Formula (4):

(4) $$i_{\mathrm{inter}}=i_{\mathrm{pol}}-i_{\mathrm{depol}}-i_{\mathrm{con}}$$

where *i_inter_* is the interfacial polarization current, *i_pol_* is the polarization current, *i_depol_* is the depolarization current and *i_con_* is the conduction current.

### 2.3. Breakdown Voltage Test

**Figure 3:**
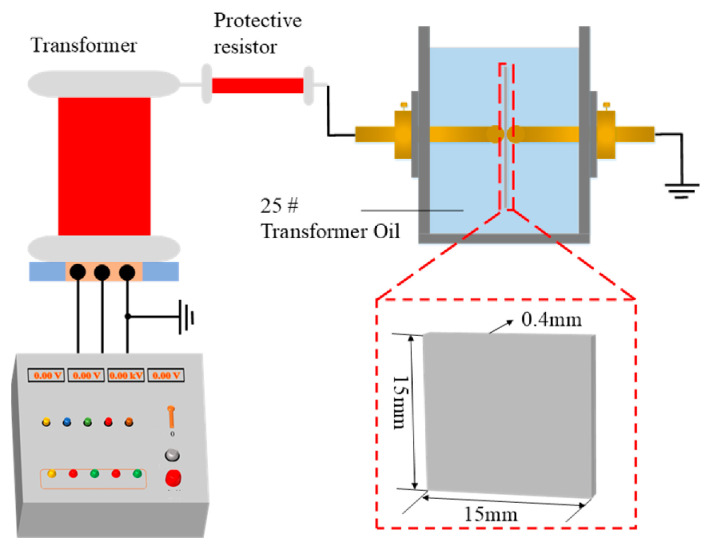
Power frequency breakdown voltage testing system [28].

Breakdown voltage tests were conducted using a sphere-sphere electrode system, as shown in Figure 3, with a sphere diameter of 25 mm. The entire electrode system was immersed in No. 25 transformer oil to effectively suppress surface flashover and prevent partial discharge before testing, ensuring that electrical breakdown occurred within the bulk of the specimen. The prepared XLPE/semiconductive shielding layer interface specimens were cut into thin sheets with dimensions of 15 mm × 15 mm × 0.4 mm for breakdown testing. Each specimen was subjected to five valid breakdown tests to obtain statistically meaningful results. During the test, the applied voltage was increased at a constant ramp rate of 1 kV/s until breakdown occurred, and the corresponding breakdown voltage was recorded.

Due to the inherent dispersion and randomness of breakdown voltage data, Weibull statistical analysis was employed., in accordance with the recommendations of the International Electrotechnical Commission (IEC) 930 and the Chinese national standard GB/T 29310-2012. A two-parameter Weibull distribution was adopted, and the voltage corresponding to a 63.2% breakdown probability is defined as the characteristic breakdown voltage. The cumulative distribution function is expressed as Formula (5):

(5) $$F\left(U,\alpha ,\beta \right)=1-\exp\left\{-{\left(\frac{U}{a}\right)}^{\beta }\right\}$$

where *F*(*U*, *α*, *β*) is the cumulative failure probability, *U* is the breakdown voltage of the specimen (kV), α is the scale parameter representing the characteristic breakdown voltage at a failure probability of 63.2% and *β* is the shape parameter describing the dispersion of breakdown voltage data.

### 2.4. Peel Adhesion Strength Test

**Figure 4:**
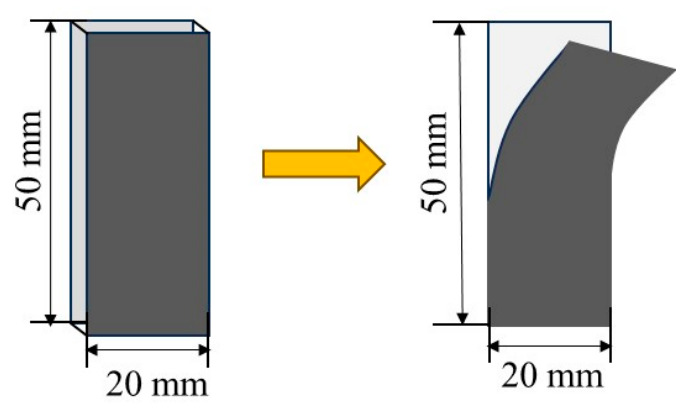
Schematic diagram of peeling at the XLPE/semiconductor layer interface.

The peel adhesion properties of the XLPE/semiconductive shielding layer interfaces prepared with different secondary crosslinking times were evaluated using a universal electronic tensile testing machine (Model 68TM-10, INSTRON, Boston, MA, USA) with a maximum load capacity of 500 N. The interface specimens were cut into strip-shaped samples, as illustrated in Figure 4 with a length of 50 mm, a width of 20 mm and an interface thickness of 0.4 mm.

The tensile tests were performed at a constant crosshead speed of 25 mm/s under ambient conditions at a temperature of (25 ± 2) °C. For each secondary crosslinking time, five specimens were tested to reduce the influence of random errors and ensure the repeatability and reliability of the experimental results.

### 2.5. Scanning Electron Microscopy Observation

To further investigate the microstructural morphology of the XLPE/semiconductive shielding layer interfaces formed under different secondary crosslinking times, scanning electron microscopy (SEM) was employed. Since the semiconductive shielding layer is a polymeric material, cutting marks produced during room temperature sectioning may adversely affect the observed SEM morphology. Therefore, cryogenic fracturing was adopted to obtain representative cross-sectional surfaces. The specimen preparation procedure was as follows.

First, specimens containing the insulation joint region were cut into strip-shaped samples, and a wedge-shaped notch was introduced along the tangential direction of the interface. Second, the specimens were immersed in liquid nitrogen and cryogenically frozen for 20 min. Third, the frozen specimens were fractured along the pre-notched region using a mechanical fixture. Finally, to improve the electrical conductivity of the semiconductive shielding layer during SEM observation, a thin and uniform gold layer was deposited on the specimen surface by ion sputtering.

In this study, a JSM-7500F field-emission scanning electron microscope produced by Japan Electronics Corporation in Tokyo, Japan was used. The instrument provides a spatial resolution ranging from1.0 nm (at 15 kV) to 1.4 nm (at 1 kV) with a magnification range from 25 to 100,000 times.

## 3. Results

### 3.1. Analysis of Polarization and Depolarization Current Results

**Figure 5:**
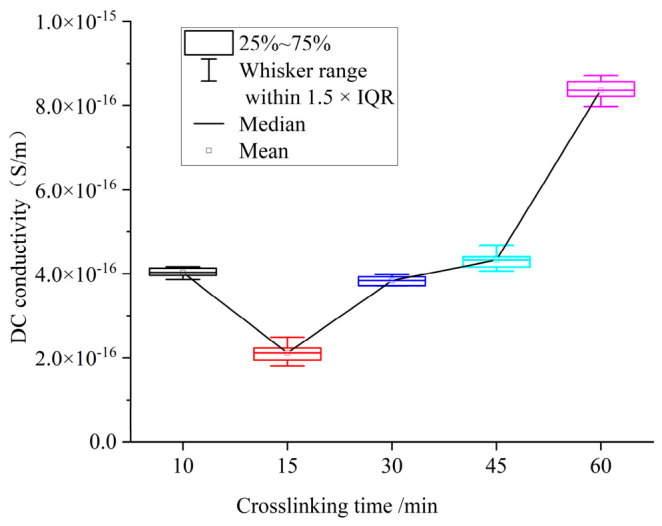
DC conductivity of the XLPE/semiconductive shielding layer interface under different secondary crosslinking times.

Based on the PDC measurement results, the DC conductivity results of the XLPE/semiconductive shielding layer were calculated using Formula (3), which are presented in Figure 5. The DC conductivity of the XLPE/semiconductive shielding layer interface exhibited an initial decrease followed by an increase with increasing secondary crosslinking time. The interfacial DC conductivity reached its minimum at a secondary crosslinking time of 15 min, indicating that the interface showed the best insulation performance under this condition. The increase became particularly pronounced at 60 min, suggesting that the interfacial insulation performance gradually deteriorated as the secondary crosslinking time increased.

The interfacial polarization current of the XLPE/semiconductive shielding layer interfaces can be used to characterize the ability of the interface to accumulate charges and respond to polarization, and its variation is closely related to the interfacial microstructural state. In general, a larger interfacial polarization current suggests that the interfacial region contains more microscopic inhomogeneities favorable for charge trapping and accumulation, such as micropores, local defects, or structural discontinuities, which usually corresponds to weaker interfacial insulation performance.

**Figure 6:**
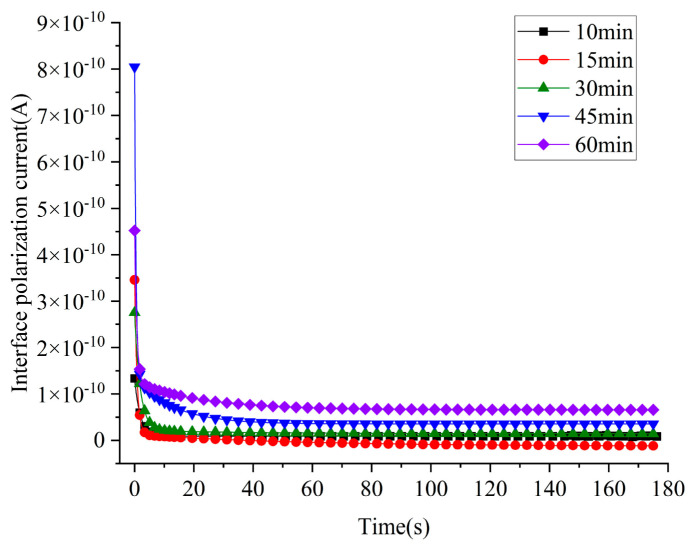
Interfacial polarization current curves of the XLPE/semiconductive shielding layer interface under different secondary crosslinking times.

The interfacial polarization current under different secondary crosslinking times was calculated using Formula (4), as shown in Figure 6. The results show that the interfacial polarization current is an initial decrease followed by an increase with increasing secondary crosslinking time, indicating that the number of defects or the degree of structural inhomogeneity in the interfacial region may show an initial decrease followed by an increase. Therefore, the charge accumulation capability exhibited an initial decrease followed by an increase.

### 3.2. Analysis of Breakdown Voltage Test Results

**Figure 7:**
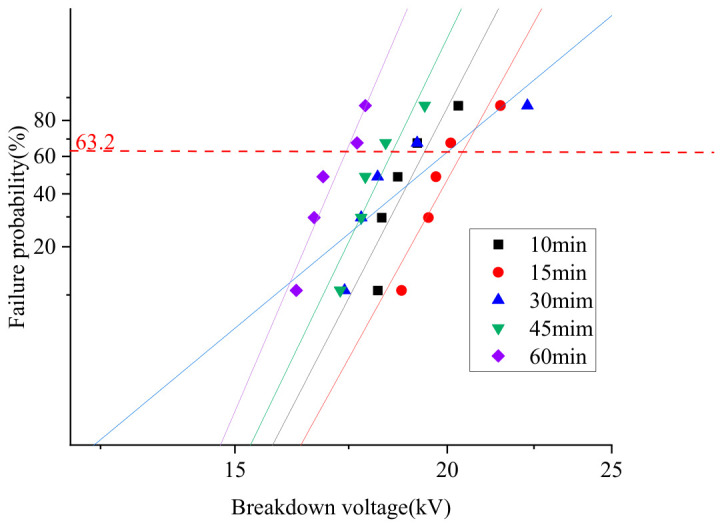
Relationship between breakdown voltage and failure probability at different secondary crosslinking times.

The Weibull distributions of breakdown voltage for the XLPE/semiconductive shielding layer interfaces with different secondary crosslinking times are shown in Figure 7 and the corresponding scale and shape parameters are summarized in Table 1. As illustrated in Figure 7 and Table 1, with increasing secondary crosslinking time, the characteristic breakdown voltage exhibited an initial increase followed by a decrease, while the shape parameter first decreased and then increased. When the secondary crosslinking time is 15 min, the characteristic breakdown voltage reaches 20.45 kV and the shape parameter is 9.57.

At a secondary crosslinking time of 60 min, the characteristic breakdown voltage decreases to the minimum value of 17.36 kV, corresponding to a reduction of 3.09 kV compared with that at 15 min. These results indicate that during the recovery process of the semiconductive shielding layer in flexible joints, excessive extension of the secondary crosslinking time can lead to a reduction in the breakdown strength of the XLPE/semiconductive shielding layer interface.

### 3.3. Analysis of Peel Adhesion Strength Test Results

**Figure 8:**
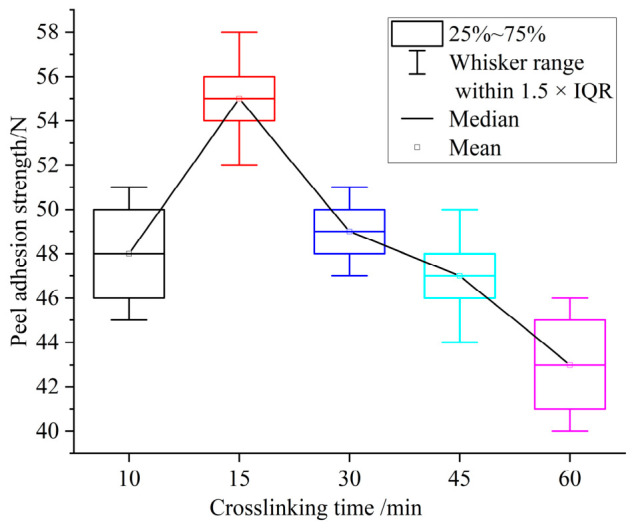
Peel adhesion strength of the XLPE/semi-conductive interface at different secondary crosslinking times.

The peel adhesion strength test results are presented in Figure 8. As the secondary crosslinking time increases, the interfacial peel adhesion strength shows an initial increase followed by a decrease. Specifically, the peel adhesion strength increases by 14.58% when the secondary crosslinking time increases from 10 min to 15 min. Then it decreases by 10.91% when the secondary crosslinking time increases from 15 min to 30 min, and by 4.08% when the secondary crosslinking time increases from 30 min to 45 min. The reduction rate of peel adhesion strength becomes progressively smaller with increasing secondary crosslinking time.

This trend indicates that prolonged secondary crosslinking time weakens the interfacial peel adhesion strength, which in turn has an adverse effect on the interfacial insulation performance.

### 3.4. Analysis of Scanning Electron Microscope Observation Results

**Figure 9:**
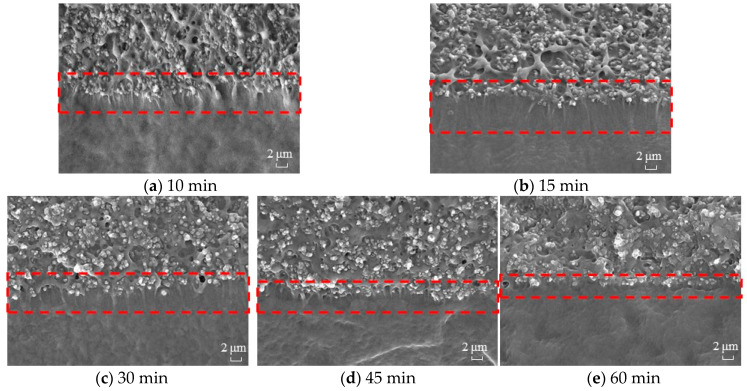
SEM images of insulation interface layer cross-sections at different secondary crosslinking times.

**Figure 10:**
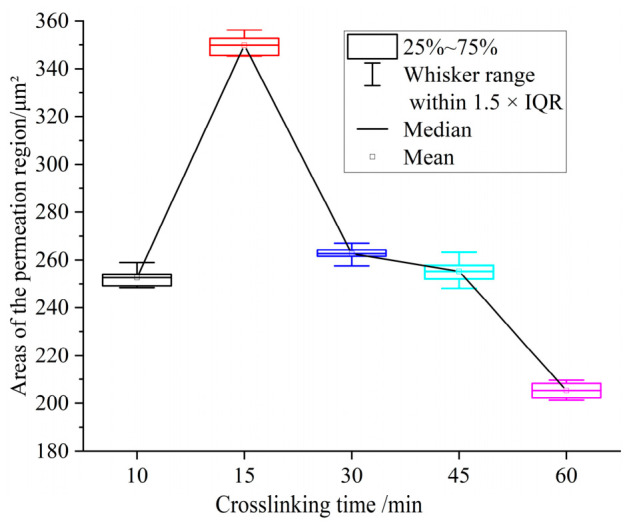
Areas of the semiconductive layer interpenetration region at different secondary crosslinking times.

After cryogenic fracturing, the fracture surfaces of XLPE/semiconductive shielding layer interface specimens prepared with different secondary crosslinking times were examined by SEM at a magnification of 15,000 times. The corresponding SEM images are shown in Figure 9a–e. As shown in Figure 9, the upper region corresponds to the semiconductive shielding layer, while the lower region corresponds to the XLPE layer. The region outlined by the red box represents the transition layer between XLPE and the semiconductive layer. To characterize the formation degree of the interfacial transition layer between XLPE and the semiconductive shielding layer, quantitative analysis was performed on the SEM images of the interfacial region. Based on the differences in surface topography between the transition layer and the adjacent materials on both sides, the upper and lower boundaries of the transition layer were identified. ImageJ software 1.x. was then used to perform pixel statistics on the enclosed region. By combining the image scale with the pixel count, the pixel area was converted into the actual area, thereby obtaining the projected area of the interfacial transition layer. The measured areas of the semiconductive layer interpenetration region are shown in Figure 10. The calculated average value in the permeation region is summarized in Table 2.

As shown in Figure 10 and Table 2, the area of the interfacial interpenetration region exhibited an initial increase followed by a decrease with increasing secondary crosslinking time, reaching its maximum at a secondary crosslinking time of 15 min. This microstructural evolution is consistent with the observed deterioration in interfacial electrical and adhesion properties, indicating that the reduction of the interpenetration region plays an important role in the degradation of interfacial performance.

## 4. Discussion

### 4.1. Relationship Between Interfacial Properties and Secondary Crosslinking Time

Secondary crosslinking time is a critical processing parameter that governs the interfacial structure of the XLPE/semiconductive shielding layer system and has a significant influence on both its electrical and mechanical properties. As the secondary crosslinking time increases, the characteristic breakdown voltage exhibits an initial increase followed by a decrease. Specifically, the breakdown voltage increases by 5.63% when the crosslinking time increases from 10 min to 15 min. Then it decreases by 3.37% when the secondary crosslinking time increases from 15 min to 30 min with the reduction further expanding to 6.07% between 30 min and 45 min, and reaching 6.47% between 45 min and 60 min. Meanwhile, the area of the interfacial interpenetration region also exhibits an initial increase followed by a decrease. with increasing secondary crosslinking time. The evolution trend of the interpenetration region is consistent with the variation trend of characteristic breakdown voltage, indicating a clear correlation between these two parameters.

The interfacial peel adhesion strength exhibits a similar dependence on secondary crosslinking time. The overall trend remains an initial increase followed by a decrease with increasing secondary crosslinking time. Interfacial peel adhesion strength fundamentally reflects the compatibility and bonding degree between the two materials at the interface. In general, an enlarged interpenetration region is beneficial for enhancing interfacial bonding. In the present study, the peel adhesion strength increases with the expansion of the interpenetration region, showing good consistency between mechanical performance and interfacial morphology. These results imply that excessive secondary crosslinking time may lead to internal structural deterioration, such as non-uniform crosslinking distribution, interfacial stress concentration or weakened chemical bonding which collectively reduce the interpenetration region area and consequently degrade the interfacial adhesion strength [29,30,31].

**Figure 11:**
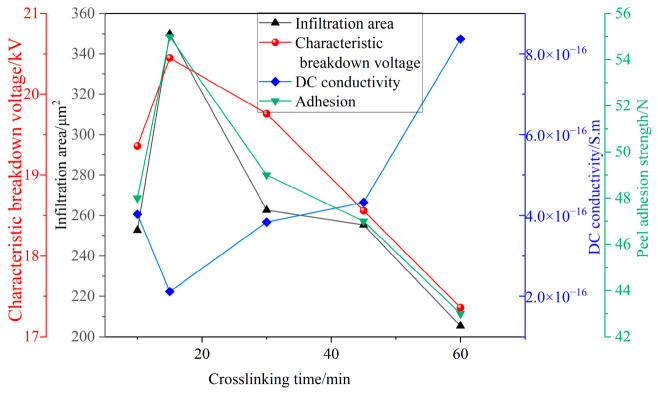
Relationship between interfacial permeation zone area and characterization parameters.

Based on the data summarized in Figure 11, the correlations among the key parameters can be further clarified. Both the characteristic breakdown voltage and the interfacial peel adhesion strength exhibit a positive correlation with the area of the interpenetration region, whereas the DC conductivity shows a negative correlation with this area. These relationships indicate that secondary crosslinking time primarily influences the overall interfacial performance by regulating the formation and evolution of the interfacial interpenetration region. As a transitional zone between the two phases, the area and structural characteristics of the interpenetration region play a decisive role in determining both the breakdown voltage and the peel adhesion strength. On the one hand, a reduction in the interpenetration region area implies a decreased effective crosslinked contact area at the XLPE/semiconductive shielding layer interface, leading to lower breakdown voltage and reduced peel adhesion strength. On the other hand, the composition and morphology of this region directly govern the interfacial insulation performance at the interface.

### 4.2. Effect of Secondary Crosslinking Time on the Interfacial Interpenetration Region

**Figure 12:**
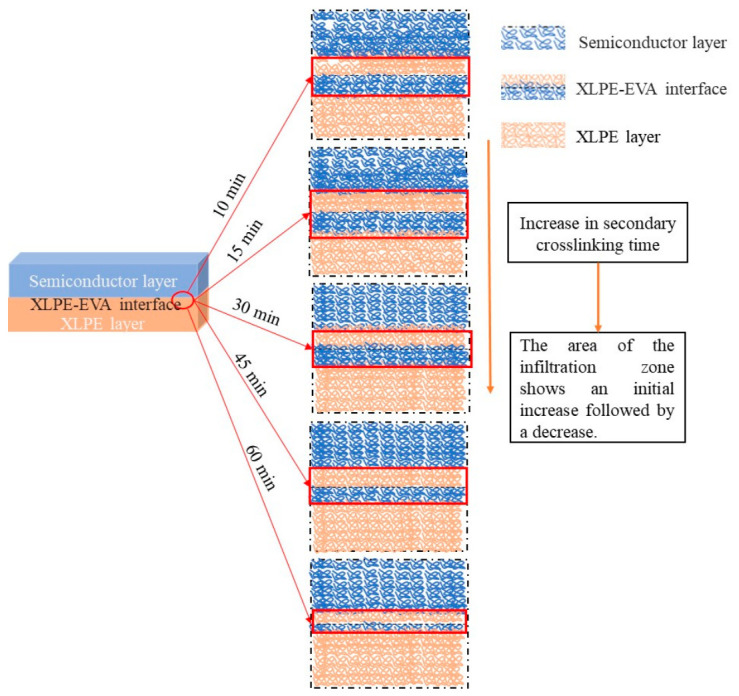
Schematic diagram of the interfacial permeation zone area at the XLPE/semiconductive shielding layer interface under different secondary crosslinking times.

From a mechanistic perspective, the formation of the interfacial interpenetration region is the result of the combined effects of multiple factors during the secondary crosslinking process. As illustrated in Figure 12, with the progression of crosslinking the interactions between fillers and the polymer matrix in the semiconductive layer, the diffusion behavior of crosslinking agents and the mobility of molecular chains at the interface all undergo continuous evolution. These coupled processes lead to the development of an interfacial region with a distinct structure and spatial gradient.

The size, uniformity and compactness of the interpenetration region not only influence the local electric field distribution near the interface but also determine the failure mode and energy dissipation mechanisms of the material under mechanical loading. A larger interpenetration region area between XLPE and EVA indicates a better secondary crosslinking effect between the two materials, resulting in higher interfacial peel adhesion strength, higher breakdown voltage, and lower DC conductivity.

Therefore, the dual effects of secondary crosslinking time on the structure of the interpenetration region must be comprehensively considered in cable insulation design and process control. By optimizing the crosslinking process, a balance can be achieved among the interpenetration region area, interfacial bonding strength and electrical performance, ultimately enhancing the overall reliability of the cable insulation system. The results of this study demonstrate that appropriate control of secondary crosslinking time plays a critical role in achieving synergistic optimization of interfacial structure and performance.

## 5. Conclusions

In this study, the effects of secondary crosslinking time on the insulation performance and microstructural characteristics of the XLPE/semiconductive shielding layer interface are systematically investigated through breakdown voltage tests, polarization and depolarization current (PDC) measurements, peel adhesion strength tests and scanning electron microscopy observations. The following conclusions have been drawn.

Excessive secondary crosslinking time significantly deteriorates the interfacial insulation performance. With increasing secondary crosslinking time, the area of the interfacial interpenetration region exhibits an initial increase followed by a decrease. The characteristic breakdown voltage also exhibits an initial increase followed by a decrease and the DC conductivity exhibits an initial decrease followed by an increase. Among the investigated conditions, a secondary crosslinking time of 15 min yields the optimal performance, characterized by the largest interpenetration region area and the highest characteristic breakdown voltage.

Secondary crosslinking time is a critical processing parameter for regulating the interfacial structure between the semiconductive shielding layer and the XLPE insulation. An appropriate secondary crosslinking time promotes interfacial diffusion and interpenetration. In contrast, excessive secondary crosslinking time suppresses the mutual interpenetration between the two phases, leading to a pronounced reduction in the interpenetration region area and consequently degrading the interfacial bonding quality and electrical performance.

As a transitional zone at the XLPE/semiconductive shielding layer interface, the size of the interpenetration region plays a decisive role in interfacial insulation performance. A larger interpenetration region contributes to the formation of a smoother dielectric gradient, alleviates electric field concentration, enhances breakdown voltage, reduces DC conductivity and ultimately optimizes the overall insulation performance.

## Figures and Tables

**Table 1:** Characteristic breakdown voltage of interface samples at different secondary crosslinking times.

Sample Type	Characteristic Breakdown Voltage/kV	Shape Parameters
10 min crosslinked sample	19.36	22.27
15 min crosslinked sample	20.45	9.57
30 min crosslinked sample	19.76	20.89
45 min crosslinked sample	18.56	23.86
60 min crosslinked sample	17.36	26.93

**Table 2:** Average values in the permeation region at different secondary crosslinking times.

Secondary Crosslinking Time/min	Interfacial Interpenetration Region/μm^2^
10	252.68
15	349.87
30	262.65
45	255.32
60	205.32

## Data Availability

The raw data supporting the conclusions of this article will be made available by the authors on request.
